# How Ciliated Protists Survive by Cysts: Some Key Points During Encystment and Excystment

**DOI:** 10.3389/fmicb.2022.785502

**Published:** 2022-02-17

**Authors:** Yuqing Li, Yurui Wang, Shijing Zhang, Xyrus X. Maurer-Alcalá, Ying Yan

**Affiliations:** ^1^Institute of Evolution and Marine Biodiversity, Ocean University of China, Qingdao, China; ^2^Laboratory of Protozoological Biodiversity and Evolution in Wetland, College of Life Sciences, Shaanxi Normal University, Xi’an, China; ^3^Division of Invertebrate Zoology, American Museum of Natural History, New York, NY, United States; ^4^Sackler Institute for Comparative Genomics, American Museum of Natural History, New York, NY, United States

**Keywords:** resting cyst, bionomic strategy, structure, factors, molecular mechanism

## Abstract

Forming cysts is a common and important bionomic strategy for microorganisms to persist in harsh environments. In ciliated protists, many species have been reported to form cysts when facing unfavorable conditions. Despite traditional studies on the morphological features of cysts and the chemical composition of cyst wall, recent research has focused more on the molecular mechanisms of encystment. The present work reviews studies on developmental features and molecular information of resting cysts in ciliates, and pays more attention to the following questions: what are the inducing factors of encystment and excystment? How does the cell change morphologically during these dynamic processes? And what molecular mechanisms underlie those changes? We also present and summarize the characteristics of cysts from diverse ciliate lineages in a phylogenetic framework, aiming to provide new perspectives for studies on adaptive evolution of unicellular eukaryotes.

## Introduction

Cyst formation, which is common in microbial organisms, is considered as an adaptive strategy against adverse environmental conditions (Corliss and Esser, 1974). The process of encystment and excystment (i.e., the E-E cycle) involves dramatic structural changes, including cell volume decrease, cyst wall (CW) formation, nuclear fusion (of some species) and ciliature resorption and regeneration (Gutiérrez et al., 1990). Additionally, the E-E cycle can differ from species to species in many aspects, for example, by the morphology of mature cysts and by the manner of escaping from the CW during excystment.

Ciliates are a hyper-diverse group of unicellular organisms and an essential component of microbial food webs (Bai et al., 2020; Ma et al., 2020; Wu et al., 2020; Zhang et al., 2020; Zhao et al., 2020, 2021; Chi et al., 2021; Wang C. et al., 2021). They are characterized by possessing two distinct types of nuclei, the somatic macronucleus and the germline micronucleus, within one cell (Prescott, 1994; Sheng et al., 2020). Despite the diverse life history, ciliates mainly reproduce sexually (i.e., conjugation) and asexually (i.e., binary fission) (Prescott, 1994; Chi et al., 2020; Gao et al., 2020). Ciliates are extremely widespread across various habitats and environmental conditions (Wang J. et al., 2021), including ephemeral vernal pools and hot springs (Kahan, 1972; Reid and John, 1983). They are capable of turning into cryptobiotic forms when facing unfavorable conditions (Beers, 1927; Repak, 1968; Gutiérrez et al., 1990; Foissner et al., 2005), among which cyst formation is a common way to engage into resting and resistant stages and to support cell dispersion (Farmer, 1980).

Research on ciliate cysts started in the mid-19th century, Claparède and Lachmann (1858) reported the first description of cysts from a shelled choreotrich ciliate *Amphorides amphora* and other tintinnids. Hereafter, the structural changes, and more recently, molecular mechanisms of the E-E cycle in ciliates have been investigated through light and electron microscopy, various staining methods, molecular techniques and ’omic analyses (e.g., Pan et al., 2019). These studies suggest that ciliates can be coaxed into cysts by various factors and the morphology of cysts vary among species (van Wagtendonk, 1955; Corliss and Esser, 1974). In addition, the expression level of related proteins might be up-regulated or down-regulated, promoting cells to form cysts or to detach from the CW during excystment as they return to suitable environments (Chen et al., 2014; Gao et al., 2015).

Given the long history of studying microorganisms in dormant stages, these stages have been reported under numerous names, such as spores, sporulation, stomatocysts, or cysts (Benítez and Gutiérrez, 1997; Foissner, 2006). The definitions given to these terms vary among studies, which means that the same word might refer to different types of cysts in different investigations. For example, forming reproductive cysts is a necessary stage in some ciliates’ life cycle (Xu et al., 2007), which is different from resting cysts. In addition, for those species undergoing asexual reproduction solely through reproductive cysts, they usually form cysts under sufficient nutrition, which is distinct from resting cyst formation under harsh environment (e.g., lack of nutrition) (Benčat’ová et al., 2020).

In the present work, we largely focus on resting cysts and choose “cyst” as the general term, which consists of resting cysts (including temporary cysts), reproductive cysts, digestive cysts, etc. We aim to summarize the basic structure, inducing factors, structural changes and responding molecular mechanisms in resting cysts.

## The Ability to Encyst

Extreme caution needs to be taken in determining whether or not a species is capable of encysting and excysting. There could be several reasons why a species has yet been reported to form cysts: (1) the species may be poorly studied, (2) the E-E cycle may rarely occur in this species. Therefore, the ability and opportunity to identify the onset of the E-E cycle are slim, especially if cyst formation is not being the species’ primary way of surviving harsh environment, (3) lastly, it is possible that this species cannot form cysts.

With the above being said, we can still review the literature and gain some hints on which ciliate groups or species are more likely to form cyst and why. For example, a number of soil-living ciliates, represented by colpodids, can transform into resting cysts promptly against desiccation, temperature fluctuations and acid (Matsuoka et al., 2017). Some ciliates achieve survival in tide pools by alternating between cysts and free-swimming forms synchronously with tides (Jonsson, 1994; Montagnes et al., 2002). Forming cysts is also essential for lineages that live in vernal pools or other environments where the pH, temperature and salinity fluctuates significantly.

## Structure of Cysts

**FIGURE 1:**
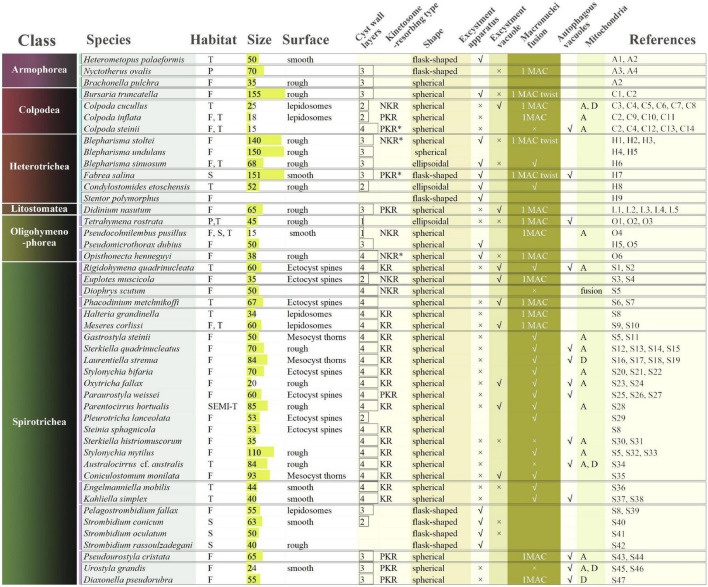
Comparison of morphological characteristics of resting cysts of 46 ciliate species representing 6 classes. T, terrestrial; P, parasitic; F, fresh water; S, sea water; SEMI-T, semi- terrestrial; A, aggregation; D, degeneration; ^∗^, classified by the present work. The detailed information of the references is shown in Supplementary Material.

To date, cysts from about 40 ciliate species have been well documented, though most studies focused predominantly on cyst morphology (Verni and Rosati, 2011; Figure 1).

**FIGURE 2:**
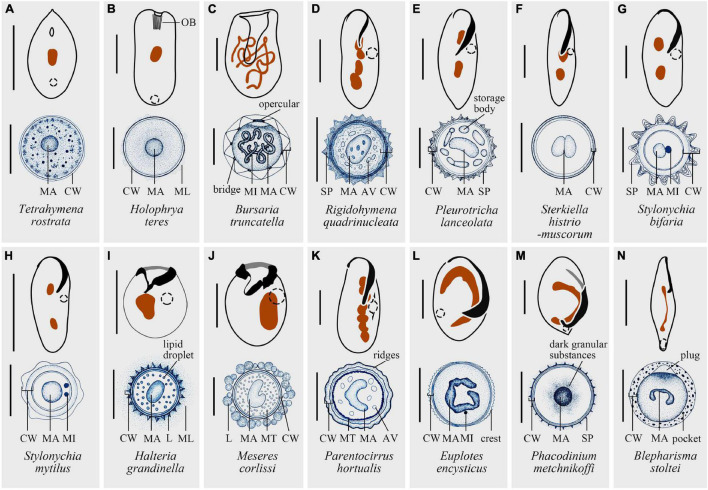
Structure diagrams of resting cysts and vegetative cells of 14 representative species. **(A)** *Tetrahymena rostrata*, modified after Stout (1954) and Zebrun et al. (1967). **(B)** *Holophrya teres*, modified after Benčat’ová et al. (2020) and Foissner (2021). **(C)** *Bursaria truncatella*: bridge, between the inner and outer cyst walls; opercular, upon emergence pore; modified after Beers (1948) and Krause and Braucker (2009). **(D)** *Rigidohymena quadrinucleata*, modified after Benčat’ová and Tirjaková (2017) and Wang J. et al. (2017). **(E)** *Pleurotricha lanceolata*: storage bodies, discontinuous ring of hyaline “lakes” in protoplast; modified after Manwell (1928), Jeffries (1956), Jeffries and Mellott (1968), and Dragesco (1972). **(F)** *Sterkiella histriomuscorum*, modified after Adl and Berger (1997), Grisvard et al. (2008) and Jiang et al. (2013). **(G)** *Stylonychia bifaria*, modified after Ricci et al. (1985), Wirnsberger et al. (1985) and Verni and Rosati (2011). **(H)** *Stylonychia mytilus*, modified after Jones (1974), Walker et al. (1975) and Zhang and Pang (1981). **(I)** *Halteria grandinella*, modified after Song (1993) and Foissner et al. (2007). **(J)** *Meseres corlissi*, modified after Petz and Foissner (1992) and Foissner et al. (2005). **(K)** *Parentocirrus hortualis*: ridges, generated by the whole cyst wall; modified after Voß (1997) and Benčat’ová et al. (2016). **(L)** *Euplotes encysticus*, modified after Rawlinson and Gates (1985), Gu and Zhang (1992), Gu and Xu (1995) and Fan et al. (2010). **(M)** *Phacodinium metchnikoffi*: dark granular substances, assumed to be the reserve products; modified after Fernandez-Galiano and Calvo (1992), de Pablo (2010) and Benčat’ová and Tirjaková (2018). **(N)** *Blepharisma stoltei*: pockets, intrinsic part of the outer wall and appearing as disk-like structures; modified after Repak and Pfister (1967), Repak (1968) and Chi et al. (2021). MA, macronucleus; CW, cyst wall; OB, oral basket; ML, mucous layer; SP, spines; AV, autophagic vacuoles; L, lepidosomes; MT, mitochondrium. Scale bars: 30 μm **(A,F,G,I,J,L)**, 50 μm **(B,D,E,K,M)**, 100 μm **(C,H,N)**.

**FIGURE 3:**
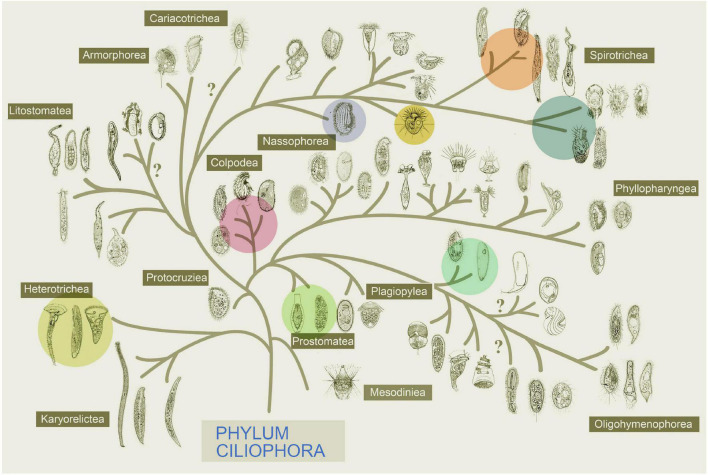
Systematic positions of the 14 species described in Figure 2, of which 5 classes within the phylum Ciliophora are represented (marked in color, modified after Gao et al., 2016).

Most cysts are spherical (Mulisch and Hausmann, 1989; Figures 2, 3) or ellipsoidal (Foissner et al., 2002), although flask-shape (Kim and Taniguchi, 1995) or disk-shaped (Gurdebeke et al., 2018) cysts have also been observed in several species. The size of cysts ranges from 15 μm in *Pseudocohnilembus pusillus* (Olendzenski, 1999) to 225 μm in *Blepharisma japonicum* (Giese, 1973). In most cases, the volume of cysts is much smaller than vegetative cells (Foissner et al., 2006) with the exception of *Strombidium oculatum* (Jonsson, 1994). Despite a few species that form colored cysts resulting from food or pigment granules, cysts of most species are colorless (Repak, 1968; Foissner et al., 2007; Benčat’ová et al., 2016; Benčat’ová and Tirjaková, 2017; Cavaleiro et al., 2018).

One of the crucial structures of cysts is the CW. CW has almost always been described as multiple layers (e.g., reviewed in Li et al., 2017), for example, two layers in *Euplotes muscicola* (Rawlinson and Gates, 1985), three layers in *Urostyla grandis* (Liu et al., 2009) and four layers in *Parentocirrus hortualis* (Benčat’ová et al., 2016). Here we follow Foissner’s (2005) extensive description of CW on *Meseres corlissi* and depict CW as five distinct layers, naming metacyst, endocyst, mesocyst, ectocyst, and pericyst, from touching the cell body to the outmost layer. Metacyst is located closest to the cell body and composed of fibrous material. Endocyst does not bear a clear structure and is the thickest layer of CW. Both mesocyst and ectocyst are built by fine fibers with different arrangement, while the scale-like lepidosomes is the main component of pericyst (Foissner, 2005). Pericyst can be adhesive, trapping bacteria as future food source or sticking to substrates and adjacent cysts (Repak and Pfister, 1967). Additionally, specialized structures like plugs (Repak and Pfister, 1967) and ornamentations were also reported (Foissner et al., 2007).

The internal structure may also differ from vegetative forms. Macronuclear fusion occurs in some multinucleated species (Rosati et al., 1983), while in other species the macronuclei twist together to squeeze into a much smaller space. Changes not only take place in the macronuclei, but also in the micronuclei, which may fuse (Zhang and Pang, 1981), degrade (Grimes, 1973a), or remain unchanged (Olendzenski, 1999).

**FIGURE 4:**
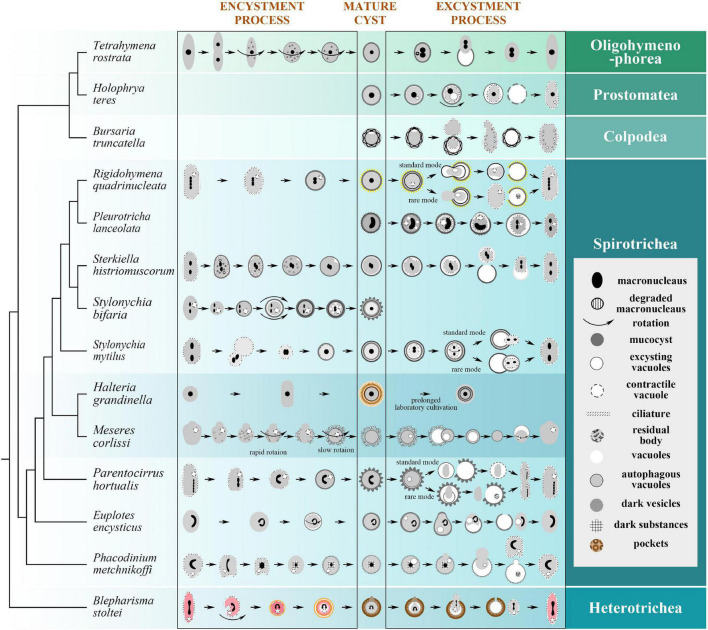
Encystment and/or excystment process of 14 representative species in Figure 2, with their phylogenetic relationships (Lynn, 2008; Berger, 2011; Gao et al., 2016; Wang J. et al., 2017; Wang et al., 2019). During the encystment process, the vegetative cells reduce in size, transform to spherical shape, absorb the ciliary structure, and secrete the CW; during the excystment process, the mature cysts restore some if not all vegetative structures within the CW prior to rupturing the CW and exiting. For the legend: macronuclei are not drawn in all stages for certain species; in *Tetrahymena rostrata*, the old macronucleus is degraded, while the new macronuclei are produced by the micronuclei; curved arrows indicate rotation and degree; mucocyst is secreted to form the cyst wall; degrading or regenerating ciliature is drawn as broken lines; vacuoles in *Parentocirrus hortualis* empty into the so-called contractile vacuole; pockets are disk-like structures consisting of unknown material. *Tetrahymena rostrata* (McArdle et al., 1980; Segade et al., 2016); *Holophrya teres* (Benčat’ová et al., 2020); *Bursaria truncatella* (Beers, 1948); *Rigidohymena quadrinucleata* (Benčat’ová and Tirjaková, 2017); *Pleurotricha lanceolata* (Jeffries, 1956); *Sterkiella histriomuscorum* (Adl and Berger, 1997); *Stylonychia bifaria* (Verni and Rosati, 2011); *Stylonychia mytilus* (Zhang and Pang, 1981); *Halteria grandinella* (Foissner et al., 2007); *Meseres corlissi* (Foissner et al., 2006; Müller, 2007); *Parentocirrus hortualis* (Benčat’ová et al., 2016); *Euplotes encysticus* (Rawlinson and Gates, 1985; Gu and Zhang, 1992; Wang B. et al., 2017); *Phacodinium metchnikoffi* (Benčat’ová and Tirjaková, 2018); *Blepharisma stoltei* (Repak, 1968).

Although there are fewer observations on the impact of encystment on other intracellular organelles, for some taxa their mitochondria may cluster or remain scattered in the mature cyst (Walker and Maugel, 1980; Verni et al., 1984). For some species, contractile vacuoles disappear, whereas autophagic vacuoles are found intact in resting cysts of many ciliates (Benčat’ová and Tirjaková, 2017; Figures 2, 4).

## Factors Required for Encystment and Excystment

### Factors Inducing Encystment

Resting cysts can not only be spontaneously generated in nature (Ricci et al., 1985; Olendzenski, 1999), but also be induced to form under laboratory conditions (Arroyo-Begovich and Cárabez-Trejo, 1982). It has been suggested that no single inducer is effective to all ciliates (Corliss and Esser, 1974), and one species may require multiple factors to form cysts (with exceptions, see below; Barker and Taylor, 1931; Johnson and Evans, 1941; van Wagtendonk, 1955).

Nutritional deficiency is a dominant factor in inducing cyst formation. This has been evident by the intracristal inclusions of mitochondria (Vickerman, 1960; Bowers and Korn, 1969), nucleolar fusion (Raikov, 1982; Frenkel, 1992), and decrease of RNA synthesis (Gutiérrez et al., 1990), found in both cysted and starved vegetive cells, indicating the connection between encystment and starvation. It is noteworthy that not only general food insufficiency can induce cyst formation, lack of specific food source, such as vitamins, can also lead to encystment (Garnjobst, 1947; van Wagtendonk, 1955; Corliss and Esser, 1974). Interstingly, in *Pelagostrombidiurn fallax*, excess food (*Rhodomonas* sp.) besides starvation can lead to cyst formation (Müller, 1996).

Other conditions, including unfavorable changes in temperature (Müller and Wünsch, 1999; Kim et al., 2002), freezing (Uspenskaya and Lozina-Lozinsky, 1979), humidity (Gutiérrez et al., 2001), salinity (Li et al., 2017), ultraviolet irradiation (Uspenskaya and Lozina-Lozinsky, 1979; Matsuoka et al., 2017), dehydration (Corliss and Esser, 1974; Gutiérrez et al., 2001), and population density (Corliss and Esser, 1974; Matsuoka, 2013; Chen et al., 2018; Sogame et al., 2019) have also been reported to be involved in inducing encystment. Additionally, change in pH (Sogame et al., 2011), concentration of Ca^2+^ or K^+^ (Yamaoka et al., 2004; Matsuoka, 2013) and oxygen (Corliss and Esser, 1974) may induce cyst formation in some species as well.

### Factors Inducing Excystment

Generally speaking, cells tend to excyst when external environment conditions become more favorable (Zhao et al., 2009; Verni and Rosati, 2011). It has been suggested that sufficient food, optimal temperature and high oxygen concentration would prompt the cells to excyst (van Wagtendonk, 1955; Müller, 2002). The optimal temperature of different species ranges widely from 19.5°C (*Gastrostyla stein*) to 35°C (*Woodrufia metabolica*) (Jeffries, 1956). Moreover, cells exposed to low light condition tend not to excyst compared to those under sufficient light (Kamiyama et al., 1995).

On one hand, certain conditions might only work for some species but not others. For instance, increased pH extends the time required for excystment in *Stylonychia pustulata* and *Pleurotricha lanceolate*, while it does not influence the process in species such as *Colpoda duodenaria* and *Didinium nasutum* (Jeffries, 1956). Whereas, in most cases, exctsyment could be induced by re-feeding (Rawlinson and Gates, 1985; Benčat’ová et al., 2020). However, the concentration and source of food is important. For example, the addition of a moderate concentration of phytoplankton facilitates excystment in tintinnid ciliates (Kamiyama, 1994), while excessive concentrations of phytoplankton inhibit excystment (Kamiyama, 1997). As for the impact of food source, Jeffries (1956) suggested that dilute plant infusions are often the best for excystment.

## Structural Changes During Encystment and Excystment

### Changes During Encystment

**FIGURE 5:**
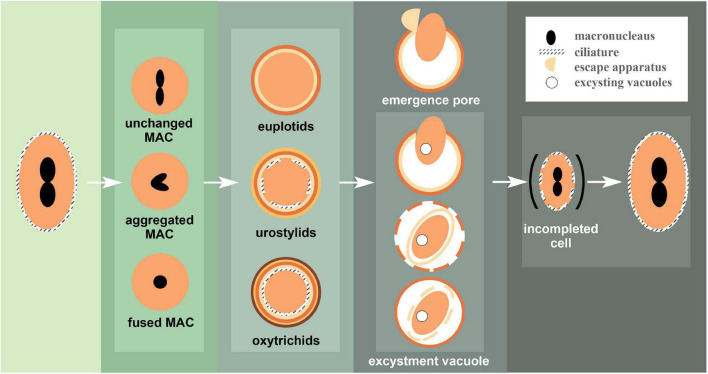
Exemplar patterns of encystment and excystment observed in ciliates. When vegetative cells encyst, the changes in macronuclear morphology are represented by three cases. Mature cysts are similarly divided into three categories based on the degree of kinetosomes resorption, which corresponds to the number of cyst wall layers. During excystment, the (in) completed vegetative cell escapes the cyst wall through an escape apparatus or under the pressure of an excystation vacuole.

The process of encystment involves changes not only in shape and the cortex (e.g., CW secretion and ciliature resorption) but also inside the cell (e.g., macronuclear aggregation, organelles clustering) among other characteristic cell recycling and remodeling processes (Walker and Maugel, 1980; Verni et al., 1984; Figures 4, 5).

Encystment generally begins with decreasing cell volume as well as transforming into spherical body shape, either due to cytoplasmic dehydration (Gutiérrez et al., 2001, 2003), or direct excretion of cytoplasmic contents (Zhang and Pang, 1981). This process leads to vast cell surface area reduction, organelle clustering (Martín-González et al., 2001), strong autophagic activity and decrease in the metabolic rate (Gutiérrez et al., 2001). In addition, Ricci et al. (1985) suggested that smaller volume requires less and thinner CW, which may be beneficial for fast CW formation and rapid response to the changing environment.

CW generation is key to the encystment process, which usually initiates after the body shape change (Figure 4). The two most intriguing questions are: (1) which part of the cell generates the CW? and (2) how is this achieved? A series of delicate works by Foissner and co-authors provided a very detailed and systematic description of CW formation in the planktonic ciliate, *Meseres corlissi* (Foissner, 2005; Foissner et al., 2005, 2006; Foissner and Pichler, 2006). The main components of the CW are the various protein complexes and carbohydrates, which usually exist in the form of chitin (Xu et al., 2007). CWs not only derive from various sources among species (e.g., precursors generated *de novo*; organelles present in the vegetative cell; combination of precursors and organelles present in the vegetative cell; without recognizable, membrane-bound precursors) (Foissner et al., 2006), but also show different origins for distinct CW layers. That’s to say, for cysts of some species, formation of the multi-layer barrier might depend on more than one secretory organelle, such as stacks of disks for ectocyst, long fibrous bodies for mesocyst, small dense bodies for the granular layers in *Oxytricha fallax* (Gutiérrez et al., 1983).

Dedifferentiation is another main feature of encystment, which is represented by resorption of the ciliature (Corliss and Esser, 1974). It is widely accepted that ciliate resting cysts are divided into three types based on the extent of ciliature resorption (Li et al., 2017): (a) non-kinetosome-resorbing cysts (NKR), with ciliary shafts (i.e., ciliary structure above kinetosomes) partially dedifferentiated (Chen et al., 2014), which appears in euplotids, nassophorean (Rawlinson and Gates, 1985), and colpodean ciliates (Tibbs, 1968); (b) partial-kinetosome-resorbing (PKR) cysts, found in urostylids and *Dileptus visscheri* whose oral and somatic ciliature disappear, while some single kinetosomes remain intact (Calvo et al., 2003); (c) kinetosome-resorbing (KR) that appears in the cysts of oxytrichids, with the absence of cilia and even of basal bodies (Grimes, 1973b). Interestingly, there is no apparent connection with ciliates’ phylogenetic position and type/extent of ciliature resorption. Among closely related taxa, ciliature resorption can be particularly different. As seen in *Australocirrus* cf. *australis*, disassembled ventral cirri were observed first, followed by the dorsal bristles (Li et al., 2017), while in *Sterkiella histriomuscorum*, dorsal kineties were found resorbed before its ventral cirri (Adl and Berger, 1997).

In addition to the changes on or above the cortex, various rearrangements have been reported to take place in the macro- and micronuclei. Besides macronuclear fusion, which is described in several species (Kamra and Sapra, 1991; Benčat’ová and Tirjaková, 2017), changes in the macronuclei include the condensation, fusion (Gutiérrez et al., 1998; Popenko et al., 1998; Martín-González et al., 2001) and extrusion (Jeffries, 1956) of chromatin, as well as the segregation, extrusion and fusion of nucleoli (Popenko et al., 1998; Martín-González et al., 2001).

Compared with macronuclei, detailed investigations of micronuclei are challenging, as micronuclei are usually too small to be clearly observed and illustrated during encystment, although Gutiérrez et al. (1998) have described that degradation and chromatin condensation occurs in micronuclei of several species. Furthermore, the numerous envelopes encapsulating micronuclei in the mature cysts may play a role in protecting the micronuclei from autophagy. It has been suggested that the extensive changes in the nuclei serve to reinforce gene-silencing and genome preservation (Gutiérrez et al., 1998).

Expendable organellar materials, such as mitochondria (Funatani et al., 2010) and ribosomes (Grimes, 1973a), can be recycled through autophagy, and new secretory organelles emerged (McArdle et al., 1980), facilitating encystment related functions. It’s suggested that the organelles and other structures in autophagic vacuoles are digested into smaller elements, so as to provide energy and materials for biosynthetic during the E-E cycle.

### Changes During Excystment

Contrary to encystment, the process of excystment aims to bring encysted cells back to their vegetative forms when facing favorable environment (Figures 4, 5).

Ciliary structures degenerate during encystment reappear when excysting. For those species with complicated and specialized ciliature (e.g., hypotrichs), the order of ciliary recovery in different cell area varies among species (Grimes, 1973a; Adl and Berger, 1997). Additionally, in some species, the ciliature is fully restored prior to the very first division after escaping from the CW [e.g., *Sterkiella histriomuscorum* (Adl and Berger, 1997)], while it can take several divisions to return to the original vegetative pattern in other species [e.g., *Parentocirrus hortualis* (Benčat’ová et al., 2016)].

Similar process occurs inside the cell. Macronuclei that have twisted or merged during encystment restore their original organization, which includes recovering the shape of the nuclei, [e.g., *Euplotes encysticus* (Rawlinson and Gates, 1985)], and the number of nuclei [e.g., *Histriculus similis* (Calvo et al., 1988) and *Stylonychia mytilus* (Zhang and Pang, 1981)]. In addition, chromatin extrusion has been observed in *Colpoda inflata*, suggesting potential changes in the macronuclear genome after excystment as well (Chessa et al., 2001). The number and distribution of mitochondria and other organelles also return to the vegetative form. For example, in *Oxytricha fallax*, the mitochondria will swell and disperse subsequently (Grimes, 1973a).

Along with the dynamic changes within the cell, the cell body eventually escapes from the CW. The escape is assisted either by a preformed apparatus (i.e., emergence pore with a removable “plug” or “operculum”) and/or a rupture generated by the pressure of cell movement inside the CW and excystation vacuole, which has been described as the contractile vacuole in *Pleurotricha lanceolata* (Jeffries, 1956). This process is typically initiated by the active rotation of the cell within the cyst, followed by lifting the plug or operculum, or generation of a rupture of a small pore (at uncertain position) on the CW. Subsequently, the cell body squeezes out through the opening and eventually regains the vegetative form after a period of free swimming.

The fate of the CW after excystment varies greatly among species: (1) the CW as a whole is left behind in the environment; (2) the encysted cell is still enclosed by the inner CW layer when breaking through the outer CW layer, afterward the inner CW layer will be ruptured and/or resorbed later, like in *Pleurotricha lanceolate* (Jeffries, 1956), *Sterkiella histriomuscorum* (Adl and Berger, 1997), *Coniculostomum monilata* (Kamra and Sapra, 1991), and *Histriculus similis* (Calvo et al., 1988); 3) all CW layers are left behind with well as the material of ruptured inner membrane (*Phacodinium metchnikoffi*, Benčat’ová and Tirjaková, 2018) in the empty resting cyst (Figure 4). The fate of the CW is not restricted to a single mode in one species. For example, both first and second types exist in *Rigidohymena quadrinucleata* (Benčat’ová and Tirjaková, 2017) and *Stylonychia mytilus* cysts (Zhang and Pang, 1981), and both second and third types have been found in *Parentocirrus hortualis* (Benčat’ová et al., 2016).

## Mechanism Involved in Encystment and Excystment

With the development of molecular biology and high throughput sequencing techniques, studies on ciliate cysts have been extended beyond morphological analyses to the exploration of the underlying molecular mechanisms. No evidence of DNA synthesis (i.e., replication) has been revealed during encystment, accompanied by extremely low levels of transcription and translation (Gutiérrez et al., 1990). However, continued protein synthesis and low-level energy metabolism led to the hypothesis that the dormant cysts are not simply “sleeping” (Chen et al., 2014). Here we summarize some important pathways, focusing on signal transduction and metabolism during the E-E cycle.

### Mechanisms Involved in Encystment

#### Signal Transduction

The first question intrigued the researchers is how the cell “senses” the environmental signal and initiates encystment. Several studies presented that the Ca^2+^/calmodulin pathway may play an important role in signal transduction during encystment (e.g., Pan et al., 2019; Matsuoka, 2021). This signaling pathway has been extensively investigated in two ciliate systems, *Pseudourostyla cristata* and *Colpoda cucullus*, which could be induced to encyst with Ca^2+^ in the culture medium (Matsuoka et al., 2009; Pan et al., 2019). Results from both systems hint that the pathway might be triggered by the increase of intracellular Ca^2+^, which could result from either an inflow from extracellular environment or a release from intracellular vesicles that store Ca^2+^ (Matsuoka et al., 2009). Shimada et al. (2021) proposed that, when *Colpoda cucullus* is induced to encyst by rapidly increase temperature, its transient receptor potential (TRP) channels may sense this stimulation, and inositol trisphosphate (IP3) will induce the release of Ca^2+^ into the cytoplasm from the endoplasmic reticulum, which is supported by the identification of IP3 receptor calcium ion channel protein in cyst wall proteins (Wang B. et al., 2017).

Subsequently, Ca^2+^ was suggested to cause an elevation of cyclic adenosine monophosphate (cAMP) concentration by activating adenylate cyclase (Matsuoka, 2021). Through transcriptome analysis, three pathways downstream of cAMP were illustrated: protein kinase A (PKA) related to protein phosphorylation, adenosine monophosphate activated protein kinase (AMPK) pathway related to autophagy, and PI3K/AKT pathway related to metabolism (Jiang et al., 2019).

It is known that PKA can elevate the phosphorylation level in certain proteins, thereby affecting the expression of those proteins. For example, when introducing cAMP into *Colpoda cucullus*, the phosphorylation level was increase in some proteins, including actin, ribosomal P0 proteins and histone H4 hyperacetylated form (Sogame et al., 2012a,^2014^). Actin is suggested crucial for the dynamic structural changes during encystment (Matsuoka, 2021). The phosphorylation of actin can lead to polymerization or depolymerization of actin filaments, eventually reflecting in the spherical shape of the mature cysts (Matsuoka, 2021). At the same time, the expression of actin and actin-binding protein were found upregulated in studied lineages during the process (Bouyer et al., 2009; Pan et al., 2019). In addition, the elevation and reorganization of F-actin promote the cell shrinkage and CW generation, respectively (Pan et al., 2019; Matsuoka, 2021).

The AMPK pathway, which could be activated by starvation, further regulates the autophagy through the mammalian target of rapamycin (mTOR) (Jiang et al., 2019) or forkhead box O (FOXO) signaling pathway (Pan et al., 2019). The FOXO signaling pathway involves in the regulation of genes related to stress response processes (e.g., autophagy and antioxidant) during cyst formation in *Pseudourostyla cristata* (Pan et al., 2019). However, it is noteworthy that AMPK seems to be differentially regulated, i.e., upregulated (Jiang et al., 2019) or downregulated (Pan et al., 2019), in different species during encystment.

#### Energy Metabolism

Like food vacuoles in vegetative cells, there are autophagy vacuoles in resting cysts, which could digest substances and create energy for cysts (Wu et al., 2004). The respiratory rate of *Colpoda steinii* decreases to a relatively low level during encystment (Tibbs and Marshall, 1969), and nearly no metabolic activity has been found in the mitochondrial membrane of resting cysts (Funatani et al., 2010; Sogame et al., 2014), which is consistent with the mitochondria aggregation (Figure 1) or decrease (Li et al., 2017).

Corliss and Esser (1974) summarized the reduction of respiration and decrease of enzyme activity in resting cysts of different ciliates. The electrophoresis experiment showed that, some important metabolic-related enzymes in resting cysts, such as ATPase, malic dehydrogenase and glutamic acid dehydrogenase, may own same composition as in vegetative cells, while their activity are drastically reduced (Chen et al., 2005).

The down-regulation of the related material biosynthesis might directly lead to the reduction of energy metabolism. For instance, the reduction of the synthesis of dihydrolipoyl dehydrogenase and isocitrate dehydrogenase involved in the TCA cycle, ATP synthase subunit beta involved in oxidative phosphorylation (Chen et al., 2018), and proteins related to glycolysis/gluconeogenesis (Jiang et al., 2019; Pan et al., 2019), indicates that metabolism of carbohydrate, lipid and amino acid are decreased. On the other hand, several energy metabolism related proteins were detected up-regulated or specifically expressed, such as lysozyme, which are thought to store energy for the cysts, and ε-trimethyllysine hydroxylase, inferred to influence the energy metabolism process (Chen et al., 2014).

### Mechanisms Involved in Excystment

Breakdown of CW is an important step during excystment process. Ubiquitin, ubiquitin carboxyl terminal hydrolase family protein and cullin family protein are speculated to participate in the degradation process (Wang B. et al., 2017). Besides, cysteine protease with different functions may also involve in the CW breakdown process or signal transduction pathway that is sensitive to ambient pH (Villalobo et al., 2003).

The homologs of dead box RNA helicases, which regulate transcription and mRNA turnover, were detected 10 min after the induction of excystment in *Colpoda* (Sogame et al., 2013), indicating the recover of transcription. Comparative transcriptome analyses of *Colpoda aspera* showed that the synthesis of palmitic acid and its synthetic substrate hexadecanoyl-CoA are both up-regulated when forming resting cysts. Palmitic acid acts as precursor to synthesize other longer fatty acids during vegetative periods, and was speculated to accumulate as fat storage to be used during excystment (Jiang et al., 2019).

Elongation factor 1α (EF-1α) is upregulated or downregulated during encystment process, and downregulated in the excystment process (Sogame et al., 2012b,^2013^; Chen et al., 2018). As a multifunctional protein, EF-1α may be involved in protein synthesis, proteasomal degradation, nuclear export, as well as actin or microtubule bundling activity (Sogame et al., 2013; Chen et al., 2018), which can partially explain the contradictory results and imply the relationship between EF-1α and the structural changes in the excystment process.

## Speculation

Forming cysts is thought to be beneficial for ciliates in several ways, including facilitating survival under adverse environment, promoting dispersion to new conditions and retarding the aging of the population. Dynamic changes presented in morphological and molecular levels are regulated by complex pathways and numerous genes are involved in the regulatory network that we are starting to understand. Intriguing questions like how the different pathways interplay and how the ciliature degenerates and regenerates are waiting to be solved. Further elucidation of the underlying mechanisms of the E-E cycle will provide more insights on reversible cell differentiation in eukaryotes.

## Author Contributions

YY contributed to conception and design of the study. YL organized the resources needed for the study. YL wrote the first draft of the manuscript. YL, YW, SZ, XM-A, and YY contributed to manuscript revision and approved the submitted version. All authors contributed to the article and approved the final version.

## Conflict of Interest

The authors declare that the research was conducted in the absence of any commercial or financial relationships that could be construed as a potential conflict of interest.

## Publisher’s Note

All claims expressed in this article are solely those of the authors and do not necessarily represent those of their affiliated organizations, or those of the publisher, the editors and the reviewers. Any product that may be evaluated in this article, or claim that may be made by its manufacturer, is not guaranteed or endorsed by the publisher.
