# Supplementary material for: How Ciliated Protists Survive by Cysts: Some Key Points During Encystment and Excystment
Source: Front Microbiol. 2022 Feb 17;13:785502. doi: 10.3389/fmicb.2022.785502 (PMC8891572; doi:10.3389/fmicb.2022.785502)
Supplement: Supplementary file 1 [file Table_1.doc]

A1, (Foissner, 2016); A2, (Bourland et al., 2018);

A3, (Lucas, 1927); A4, (Lucas, 1928);

A2, (Bourland et al., 2018);

C1, (Beers, 1948); C2, (Foissner, 1993);

C3 (Goodey, 1913); C4 (Martín-González et al., 1991); C5, (Kida and Matsuoka, 2006); C6, (Funatani et al., 2010); C7, (Funadani et al., 2013); C8, (Sogame et al., 2019);

C2, (Foissner, 1993); C9, (Martín-Gonzalez et al., 1992); C10, (Chessa et al., 1994); C11, (Kassab et al., 2002);

C2, (Foissner, 1993); C4, (Martín-González et al., 1991); C12, (Tibbs, 1968); C13, (Ruthmann and Kuck, 1985); C14, (Shatilovich et al., 2015);

H1, (Repak and Pfister, 1967); H2, (Repak, 1968); H3, (Walsh and Isquith, 1979);

H4, (Giese, 1973); H5, (Mulisch and Hausmann, 1989);

H6, (Cavaleiro et al., 2018);

H7, (Repak and Anderson, 1990);

H8, (Foissner et al., 2002);

H9, (Reid and John, 1983);

L1, (Beers, 1935); L2, (Beers, 1945); L3, (Dippell and Grimes, 1966); L4, (Holt and Chapman, 1971); L5, (Heydarnejad, 2008);

O1, (Stout, 1954); O2, (Zebrun et al., 1967); O3, (McArdle et al., 1980);

O4, (Olendzenski, 1999);

H5, (Mulisch and Hausmann, 1989); O5, (Arroyo-Begovich and Carabez-Trejo, 1982);

O6, (Calvo et al., 2003)

S1, (Benčaťová and Tirjaková, 2017); S2, (Wang et al., 2017);

S3, (Rawlinson and Gates, 1985); S4, (Gu and Xu, 1995);

S5, (Walker and Maugel, 1980);

S6, (de Pablo, 2010); S7, (Benčaťová and Tirjaková, 2018);

S8, (Foissner et al., 2007);

S9, (Foissner et al., 2006); S10, (Müller, 2007);

S5, (Walker and Maugel, 1980); S11, (Gutiérrez, 1985);

S12, (Calvo et al., 1983); S13, (Calvo et al., 1986); S14, (Calvo et al., 1988); S15, (Berger, 1999);

S16, (Gutiérrez et al., 1981); S17, (Gutiérrez and Pérez-Silva, 1983); S18, (Gutiérrez et al., 1983a); S19, (Gutiérrez et al., 1983b);

S20, (Verni et al., 1984); S21, (Ricci et al., 1985); S22, (Verni and Rosati, 2011);

S23, (Hashimoto, 1963); S24, (Grimes, 1973);

S25, (Heumann, 1975); S26, (Delgado et al., 1987); S27, (Gu et al., 1999);

S28, (Benčaťová et al., 2016);

S29, (Jeffries, 1956);

S8, (Foissner et al., 2007);

S30, (Adl and Berger, 1997); S31, (Grisvard et al., 2008);

S5, (Walker and Maugel, 1980); S32, (Walker et al., 1975); S33, (Zhang and Pang, 1981);

S34, (Li et al., 2017);

S35, (Kamra and Sapra, 1991);

S36, (Wirnsberger-Aescht et al., 1990);

S37, (Foissner and Foissner, 1986); S38, (Foissner and Foissner, 1987);

S8, (Foissner et al., 2007); S39, (Müller, 1996);

S40, (Kim and Taniguchi, 1995);

S41, (Montagnes et al., 2002);

S42, (McManus et al., 2018);

S43, (Gu et al., 2002); S44, (Pan et al., 2019);

S45, (Rios et al., 1985); S46, (Zhao et al., 2009);

S47, (Sun et al., 2014);

Adl, S. M., and Berger, J. D. (1997). Timing of life cycle morphogenesis in synchronous samples of *Sterkiella histriomuscorum*. 1. The vegetative cell cycle. *Eur. J. Protistol.* 33, 99–109. doi: 10.1016/S0932-4739(97)80025-9

Arroyo-Begovich, A., and Carabez-Trejo, A. (1982). Location on chitin in the cyst wall of *Entamoeba invadens* with colloidal gold tracers. *J. Parasitol.* 68, 253–258.

Beers, C. D. (1935). Structural changes during encystment and excystment in the ciliate *Didinium nasutum*. *Arch. Protist.* 84, 133–155.

Beers, C. D. (1945). The excystment process in the ciliate *Didinium nasutum*. *J. Elisha Mitchell Sci. Soc.* 61, 264–275.

Beers, C. D. (1948). Excystment in the ciliate *Bursaria truncatella*. *Biol. Bull.* 94, 86–98.

Benčaťová, S., and Tirjaková, E. (2017). A study on resting cysts of an oxytrichid soil ciliate, *Rigidohymena quadrinucleata* (Dragesco and Njine, 1971) Berger, 2011 (Ciliophora, Hypotrichia), including notes on its encystation and excystation process. *Acta Protozool.* 56, 77–91. doi: 10.4467/16890027AP.17.007.7482

Benčaťová, S., and Tirjaková, E. (2018). Light microscopy observations on the encystation and excystation processes of the ciliate *Phacodinium metchnikoffi* (Ciliophora, Phacodiniidae), including additional information on its resting cysts structure. *Biologia (Bratisl).* 73, 467–476. doi: 10.2478/s11756-018-0059-9

Benčaťová, S., Tirjaková, E., and Vďačný, P. (2016). Resting cysts of *Parentocirrus hortualis* Voss, 1997 (Ciliophora, Hypotrichia), with preliminary notes on encystation and various types of excystation. *Eur. J. Protistol.* 53, 45–60. doi: 10.1016/j.ejop.2015.12.003

Berger, H. (1999). Monograph of the Oxytrichidae (Ciliophora, Hypotrichia). *Monogr. Biol.* 78, 1–1080. doi: 10.1007/978-94-011-4637-1

Bourland, W., Rotterova, J., and Cepicka, I. (2018). Morphologic and molecular characterization of *Brachonella pulchra* (Kahl, 1927) comb. nov (Armophorea, Ciliophora) with comments on cyst structure and formation. *Int. J. Syst. Evol. Microbiol.* 68, 3052–3065. doi: 10.1099/ijsem.0.002888

Calvo, P., Fernandez-Aliseda, M. C., Garrido, J., and Torres, A. (2003). Ultrastructure, encystment and cyst wall composition of the resting cyst of the peritrich ciliate *Opisthonecta henneguyi*. *J. Eukaryot. Microbiol.* 50, 49–56. doi: 10.1111/j.1550-7408.2003.tb00105.x

Calvo, P., Martin, J., Delgado, P., and Torres, A. (1988). Cortical morphogenesis during excystment in *Histriculus similis* (Hypotrichida: Oxytrichidae). *J. Protozool.* 35, 177–181. doi: 10.1111/j.1550-7408.1988.tb04320.x

Calvo, P., Torres, A., Navas, P., and Perez-Silva, J. (1983). Complex carbohydrates in the cyst wall of *Histriculus similis*. *Microbiology* 129, 829–832. doi: 10.1099/00221287-129-3-829

Calvo, P., Torres, A., and Perez-Silva, J. (1986). Ultrastructural and cytochemical study of the encystment in the hypotrichous ciliate *Histriculus similis*. *Arch. Protistendk.* 132, 201–211. doi: 10.1016/S0003-9365(86)80035-5

Cavaleiro, J., Fernandes, N. M., da Silva-Neto, I. D., and Soares, C. A. G. (2018). Resting cysts of the pigmented ciliate *Blepharisma sinuosum* Sawaya, 1940 (Ciliophora: Heterotrichea). *J. Eukaryot. Microbiol.* 65, 422–426. doi: 10.1111/jeu.12483

Chessa, M. G., U., D. C. M., M., C., and P., P. (1994). “Ultrastructural cortical aspects of cell differentiation during long-term resting encystment in *Colpoda inflata*,” in *Contribution to animal biology*. eds. R. Argano, C. Cirotto, E. G. Milano, and L. Masfrolia (Palermo: Halocynthia Association), 169–172.

de Pablo, P. C. (2010). *Caracterización filogenética de Phacodinium metchnikoffi: Análisis comparativo de datos morfológicos, morfogenéticos y moleculares.* Universidad Comlutense de Madrid.

Delgado, P., Calvo, P., and Torres, A. (1987). Encystment in the hypotrichous ciliate *Paraurostyla weissei* : ultrastructure and cytochemistry. *J. Protozool.* 34, 104–110. doi: 10.1111/j.1550-7408.1987.tb03142.x

Dippell, R. V., and Grimes, G. W. (1966). Occurrence of basal bodies in cortex of active and encysted *Didinium nasutum*. *J. Protozool.* 13 (suppl.), 9.

Foissner, I., and Foissner, W. (1986). *Ciliomyces spectabilis*, nov. gen., nov. spec., a zoosporic fungus which parasitizes cysts of the ciliate *Kahliella simplex*. *Z. Parasitenkunde* 72, 29–41.

Foissner, I., and Foissner, W. (1987). The fine structure of the resting cysts of *Kahliella simplex* (Ciliata, Hypotrichida). *Zool. Anz.* 218, 65–74.

Foissner, W. (1993). “Colpodea (Ciliophora),” in *Protozoenfauna*. ed. D. Matthes (Stuttgart, Jena, New York: Gustav Fischer Verlag), 798.

Foissner, W. (2016). *Heterometopus meisterfeldi* nov gen., nov spec. (Protozoa, Ciliophora), a new metopid from Australia. *Eur. J. Protistol.* 55, 118–127. doi: 10.1016/j.ejop.2015.11.005

Foissner, W., Agatha, S., and Berger, H. (2002). *Soil ciliates (Protozoa, Ciliophora) from Namibia (Southwest Africa), with emphasis on two contrasting environments, the Etosha region and the Namib Desert.* Denisia.

Foissner, W., Müller, H., and Agatha, S. (2007). A comparative fine structural and phylogenetic analysis of resting cysts in oligotrich and hypotrich Spirotrichea (Ciliophora). *Eur. J. Protistol.* 43, 295–314. doi: 10.1016/j.ejop.2007.06.001

Foissner, W., Pichler, M., Al-Rasheid, K. A. S., and Weisse, T. (2006). The unusual, lepidosome-coated resting cyst of *Meseres corlissi* (Ciliophora: Oligotrichea): encystment and genesis and release of the lepidosomes. *Acta Protozool.* 45, 323–338. doi: 10.1186/1475-2859-5-32

Funadani, R., Suetomo, Y., and Matsuoka, T. (2013). Emergence of the terrestrial ciliate *Colpoda cucullus* from a resting cyst: rupture of the cyst wall by active expansion of an excystment vacuole. *Microbes Environ.* 28, 149–152. doi: 10.1264/jsme2.ME12145

Funatani, R., Kida, A., Watoh, T., and Matsuoka, T. (2010). Morphological events during resting cyst formation in the ciliate *Colpoda cucullus*. *Protistology* 6, 204–217.

Giese, A. C. (1973). *Blepharisma: The Biology of a Light Sensitive Protozoa.* Stanford: Stanford University Press.

Goodey, T. (1913). The excystation of *Colpoda cucullus* from its resting cysts, and the nature and properties of the cyst membranes. *Proc. Roy. Soc* 86, 427–439. doi: 10.1098/rspb.1913.0041

Grimes, G. W. (1973). Differentiation during encystment and excystment in *Oxytricha fallax*. *J. Protozool.* 20, 92–104. doi: 10.1111/j.1550-7408.1973.tb06009.x

Grisvard, J., Lemullois, M., Morin, L., and Baroin-Tourancheau, A. (2008). Differentially expressed genes during the encystment–excystment cycle of the ciliate *Sterkiella histriomuscorum*. *Eur. J. Protistol.* 44, 278–286. doi: 10.1016/j.ejop.2008.02.003

Gu, F., Ni, B., Ji, L., and Sui, S. (1999). Some ultrastructural studies on resting cysts and their organelles in *Paraurostyla weissei* (Ciliophora, Hypotrichida). *Zool. Res.* 20, 406–410. doi: 10.3321/j.issn:0254-5853.1999.06.002

Gu, F., Ni, B., Yang, Z., and Du, B. (2002). Ultrastructure of the vegetative cell and resting cyst in *Pseudourostyla cristat* (Ciliophora, Hypotrichida)(in Chinese). *Chin. J. Zool.* 48, 251–257.

Gu, F., and Xu, J. (1995). A TEM study on pre-excystment cellular structures of *Euplotes encysticus*. *Cell Res.* 5, 125–133. doi: 10.1038/cr.1995.12

Gutiérrez, J. C. (1985). Quantitative cytochemical study of chromatin and histones on isolated macronuclear masses from the resting cysts of *Gastrostyla steinii*. *Microbios* 43, 43–51.

Gutiérrez, J. C., and Pérez-Silva, J. (1983). Ultrastructural aspects of the precystic and cystic cytoplasm of the hypotrichous ciliate, *Laurentiella acuminata*. *Acta Protozool.* 22, 203–210.

Gutiérrez, J. C., Torres, A., and Pérez-Silva, J. (1981). Excystment cortical morphogenesis and nuclear processes during encystment and excystment in *Laurentiella acuminata* (Hypotrichida Oxytrichidae). *Acta Protozool.* 20.

Gutiérrez, J. C., Torres, A., and Pérez-Silva, J. (1983a). Fine structure of the cyst wall of *Laurentiella acuminata* (Hypotrichida: Oxytrichidae). *Trans. Am. Microsc. Soc* 102, 55–59. doi: 10.2307/3225925

Gutiérrez, J. C., Torres, A., and Pérez-Silva, J. (1983b). Structure of cyst wall precursors and kinetics of their appearance during the encystment of *Laurentiella acuminata* (Hypotrichida, Oxytrichidae). *J. Protozool.* 30, 226–233. doi: 10.1111/j.1550-7408.1983.tb02908.x

Hashimoto, K. (1963). Formation of ciliature in excystment and induced re‐encystment of *Oxytricha fallax* Stein. *J. Protozool.* 10, 156–166. doi: 10.1111/j.1550-7408.1983.tb02908.x

Heumann, J. (1975). Conjugation in the hypotrich ciliate, *Paraurostyla weissei* (Stein): a scanning electron microscope study. *J. Protozool.* 22, 392–397. doi: 10.1111/j.1550-7408.1975.tb05189.x

Heydarnejad, M. S. (2008). Effects of photoperiod and temperature on *Didinium nasutum* encystment. *Malays. Appl. Biol.* 37, 29–33.

Holt, P. A., and Chapman, G. B. (1971). The fine structure of the cyst wall of the ciliated protozoon *Didinium nastum*. *J. Protozool.* 18, 604–614. doi: 10.1111/j.1550-7408.1971.tb03384.x

Jeffries, W. B. (1956). Studies on excystment in the hypotrichous ciliate *Pleurotricha lanceolata*. *J. Protozool.* 3, 136–144. doi: 10.1111/j.1550-7408.1956.tb02448.x

Kamra, K., and Sapra, G. R. (1991). Formation of a transient corticotype during excystment in *Coniculostomum monilata* (Oxytrichidae, Hypotrichida) and its modification by reorganizations. *Eur. J. Protistol.* 27, 331–342. doi: 10.1016/S0932-4739(11)80249-X

Kassab, K., Ben Amor, T., Jori, G., and Coppellotti, O. (2002). Photosensitization of *Colpoda inflata* cysts by meso-substituted cationic porphyrins. *Photoch. Photobio. Sci.* 1, 560–564. doi: 10.1039/B201267G

Kida, A., and Matsuoka, T. (2006). Cyst wall formation in the ciliated protozoan *Colpoda cucullus*: cyst wall is not originated from pellicle membranes. *Invert. Surviv. J.* 3, 77–83. doi: 10.1016/j.ejheart.2008.05.014

Kim, Y. O., and Taniguchi, A. (1995). Excystment of the oligotrich ciliate *Strombidium conicum*. *Aquat. Microb. Ecol.* 9, 149–156. doi: 10.3354/ame009149

Li, Q., Sun, Q., Fan, X., Wu, N., Ni, B., and Gu, F. (2017). The differentiation of cellular structure during encystment in the soil hypotrichous ciliate *Australocirrus* cf. *australis* (Protista, Ciliophora). *Anim. Cells Syst.* 21, 45–52. doi: 10.1080/19768354.2016.1262896

Lucas, C. L. T. (1927). Two new species of amoeba found in cockroaches: with notes on the cysts of *Nyctotherus ovalis* Leidy. *Parasitology* 19, 223–235. doi: 10.1017/S003118200000562X

Lucas, C. L. T. (1928). A study of excystation in *Nyctotherus ovalis*: with notes on other intestinal protozoa of the cockroach. *J. Parasitol.* 14, 161–176. doi: 10.2307/3271996

Müller, H. (1996). Encystment of the freshwater ciliate *Pelagostrombidium fallax* (Ciliophora, Oligotrichida) in laboratory culture. *Aquat. Microb. Ecol.* 11, 289–295. doi: 10.3354/ame011289

Müller, H. (2007). Live observation of excystment in the spirotrich ciliate *Meseres corlissi*. *Eur. J. Protistol.* 43, 95–100. doi: 10.1016/j.ejop.2006.11.003

Martín-González, A., Benítez, L., and Gutiérrez, J. C. (1991). Cortical and nuclear events during cell division and resting cyst formation in *Colpoda inflata*. *J. Protozool.* 38, 338–344. doi: 10.1111/j.1550-7408.1991.tb01370.x

Martín-Gonzalez, A., Benitez, L., Palacios, G., and Gutiérrez, J. C. (1992). Ultrastructural analysis of resting cysts and encystment in *Colpoda inflata.* 1. Normal and abnormal resting cysts. *Cytobios* 72, 7–18.

McArdle, E. W., Bergquist, B. L., and Ehret, C. F. (1980). Structural changes in *Tetrahymena rostrata* during induced encystment. *J. Protozool.* 27, 388–397. doi: 10.1111/j.1550-7408.1980.tb05382.x

McManus, G. B., Liu, W. W., Cole, R. A., Biemesderfer, D., and Mydosh, J. L. (2018). *Strombidium rassoulzadegani*: a model species for chloroplast retention in Oligotrich ciliates. *Front. Mar. Sci.* 5. doi: 10.3389/Fmars.2018.00205

Montagnes, D. J. S., Lowe, C., Poulton, A., and Jonsson, P. R. (2002). Redescription of *Strombidium oculatum* Gruber 1884 (Ciliophora, Oligotrichia). *J. Eukaryot. Microbiol.* 49, 329–337. doi: 10.1111/j.1550-7408.2002.tb00379.x

Mulisch, M., and Hausmann, K. (1989). Localization of chitin on ultrathin sections of cysts of two ciliated protozoa, *Blepharisma undulans* and *Pseudomicrothorax dubius*, using colloidal gold conjugated wheat germ agglutinin. *Protoplasma* 152, 77–86. doi: 10.1007/BF01323065

Olendzenski, L. C. (1999). Growth, fine structure and cyst formation of a microbial mat ciliate: *Pseudocohnilembus pusillus* (Ciliophora, scuticociliatida). *J. Eukaryot. Microbiol.* 46, 132–141. doi: 10.1111/j.1550-7408.1999.tb04596.x

Pan, N., Niu, T., Bhatti, M. Z., Zhang, H., Fan, X., Ni, B., et al. (2019). Novel insights into molecular mechanisms of *Pseudourostyla cristata* encystment using comparative transcriptomics. *Sci. Rep.* 9. doi: 10.1038/S41598-019-55608-7

Rawlinson, N. G., and Gates, M. A. (1985). The excystment process in the ciliate *Euplotes muscicola*: an integrated light and scanning electron microscopic study. *J. Protozool.* 32, 729–735. doi: 10.1111/j.1550-7408.1985.tb03109.x

Reid, P. C., and John, A. W. G. (1983). Resting cysts in the ciliate class polyhymenophorea: phylogenetic implications. *J. Protozool.* 30, 710–713. doi: 10.1111/j.1550-7408.1983.tb05348.x

Repak, A. J. (1968). Encystment and excystment of the heterotrichous ciliate *Blepharisma stoltei* Isquith. *J. Protozool.* 15, 407–412. doi: 10.1111/j.1550-7408.1968.tb02148.x

Repak, A. J., and Anderson, O. R. (1990). The fine structure of the encysting salt marsh heterotrich ciliate *Fabrea salina*. *J. Morphol.* 205, 335–341. doi: 10.1002/jmor.1052050308

Repak, A. J., and Pfister, R. M. (1967). Electron microscopical observations on the extracellular structures of the resting cyst of *Blepharisma stoltei*. *Trans. Amer. Micros. Soc.*, 417–421. doi: 10.2307/3224264

Ricci, N., Verni, F., and Rosati, G. (1985). The cyst of *Oxytricha bifaria* (Ciliata: Hypotrichida). I. Morphology and significance. *Trans. Amer. Micros. Soc.*, 70–78. doi: 10.2307/3226358

Rios, R. M., Torres, A., Calvo, P., and Fedriani, C. (1985). The cyst of *Urostyla grandis* (Hypotrichida: Urostylidae): ultrastructure and evolutionary implications. *Protistologica* 21, 481–485.

Ruthmann, A., and Kuck, A. (1985). Formation of the cyst wall of the ciliate *Colpoda steinii*. *J. Protozool.* 32, 677–682. doi: 10.1111/j.1550-7408.1985.tb03101.x

Shatilovich, A., Stoupin, D., and Rivkina, E. (2015). Ciliates from ancient permafrost: assessment of cold resistance of the resting cysts. *Eur. J. Protistol.* 51, 230–240. doi: 10.1016/j.ejop.2015.04.001

Sogame, Y., Saito, R., Sakai, T., Shimizu, T., Ono, T., Koizumi, R., et al. (2019). Lepidosomes acquire fluorescence after encystation: Including additional notes of morphological events during encystation and reconsideration of the morphological features in the ciliate *Colpoda cucullus*. *J. Protozool. Res.* 29, 51–62. doi: 10.32268/jprotozoolres.29.1-2_51

Stout, J. D. (1954). The ecology, life history and parasitism of *Tetrahymena* [Paraglaucoma] *rostrata* (Kahl) Corliss. *J. Protozool.* 1, 211–215. doi: 10.1111/j.1550-7408.1954.tb00819.x

Sun, L., Yun, M., Gu, F., and Fan, X. (2014). Observation on the ultrastructure of vegetative cells and resting cysts of *Diaxonella pseudorubra* (Ciliophora). *J. Fudan Univ. (Natural Science)* 53, 344–349. doi: 10.15943/j.cnki.fdxb-jns.2014.03.008

Tibbs, J. (1968). Fine structure of *Colpoda steinii* during encystment and excystment. *J. Protozool.* 15, 725–732. doi: 10.1111/j.1550-7408.1968.tb02202.x

Verni, F., and Rosati, G. (2011). Resting cysts: a survival strategy in Protozoa Ciliophora. *Ital. J. Zool.* 78, 134–145. doi: 10.1080/11250003.2011.560579

Verni, F., Rosati, G., and Ricci, N. (1984). The cyst of *Oxytricha bifaria* (Ciliata Hypotrichida). II: The ultrastructure. *Protistologica* 20, 87–95.

Walker, G. K., and Maugel, T. K. (1980). Encystment and excystment in hypotrich ciliates II. *Diophrys scutum* and remarks on comparative features. *Protistologica* 16, 525–531.

Walker, G. K., Maugel, T. K., and Goode, D. (1975). Some ultrastructural observations on encystment in *Stylonychia mytilus* (Ciliophora: Hypotrichida). *Trans. Amer. Micros. Soc.* 94, 147–154. doi: 10.2307/3225545

Walsh, A. W., and Isquith, I. R. (1979). Scanning electron microscopy of cysts of the ciliate *Blepharisma stoltei*. *Trans. Amer. Micros. Soc.* 98, 261–264. doi: 10.2307/3226028

Wang, J., Li, L., Warren, A., and Shao, C. (2017). Morphogenesis and molecular phylogeny of the soil ciliate *Rigidohymena quadrinucleata* (Dragesco and Njine, 1971) Berger, 2011 (Ciliophora, Hypotricha, Oxytrichidae). *Eur. J. Protistol.* 60, 1–12. doi: 10.1016/j.ejop.2017.04.006

Wirnsberger-Aescht, E., Foissner, W., and Foissner, I. (1990). Natural and cultured variability of *Engelmanniella Mobilis* (Ciliophora, Hypotrichida); with notes on the ultrastructure of its resting cyst. *Arch. Protistendk.* 138, 29–49. doi: 10.1016/S0003-9365(11)80208-3

Zebrun, W., Corliss, J. O., and Lom, J. (1967). Electron microscopical observations on the mucocysts of the ciliate *Tetrahymena rostrata*. *Trans. Amer. Micros. Soc.* 86, 28–36. doi: 10.2307/3224421

Zhang, Z., and Pang, Y. (1981). A study of the process of encystment and excystment of *Stylonychia mytilus* (in Chinese). *J. East China Norm. Univ. (Nat. Sci.)*, 113–119.

Zhao, L., Li, Y., Li, J., and Gu, F. (2009). Some ultrastructural observations of the vegetative, resting and excysting ciliate, *Urostyla grandis* (Urostylidae, Hypotrichida). *Biol. Res.* 42, 395–401. doi: 10.4067/S0716-97602009000400001
